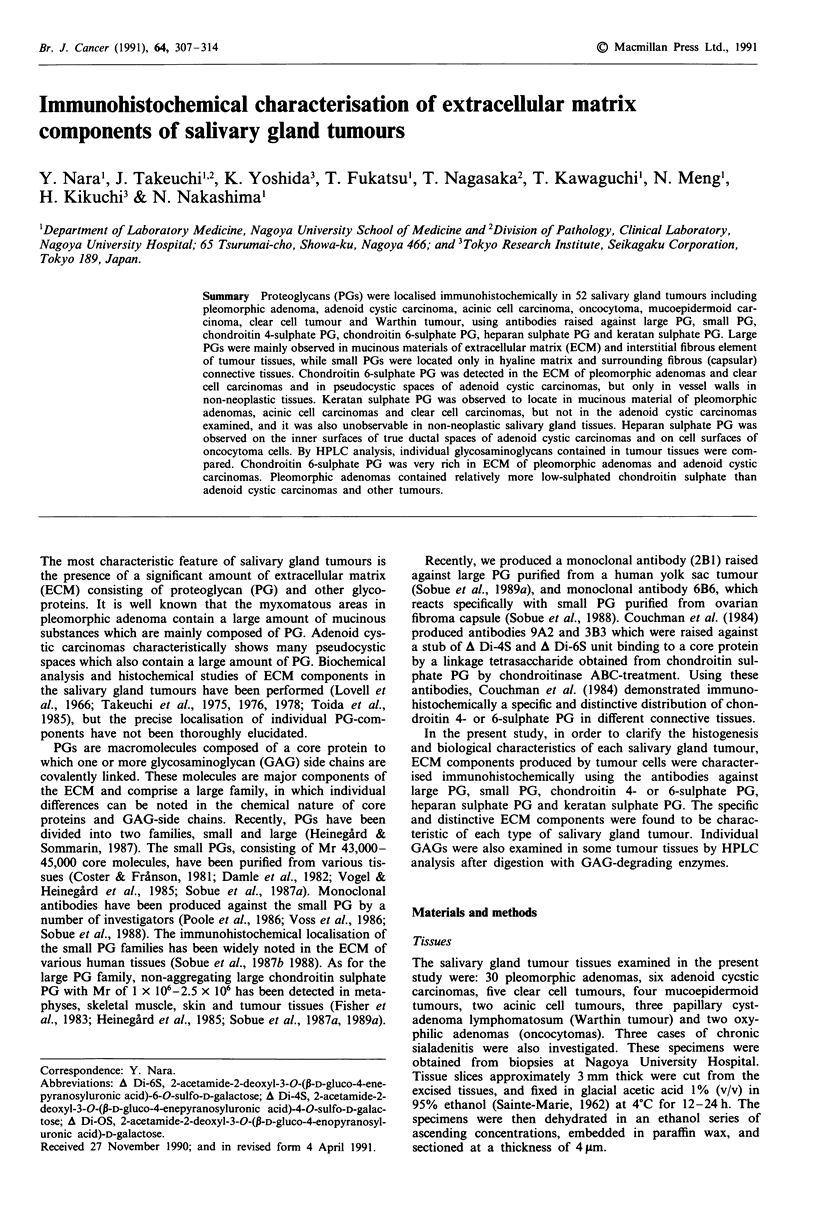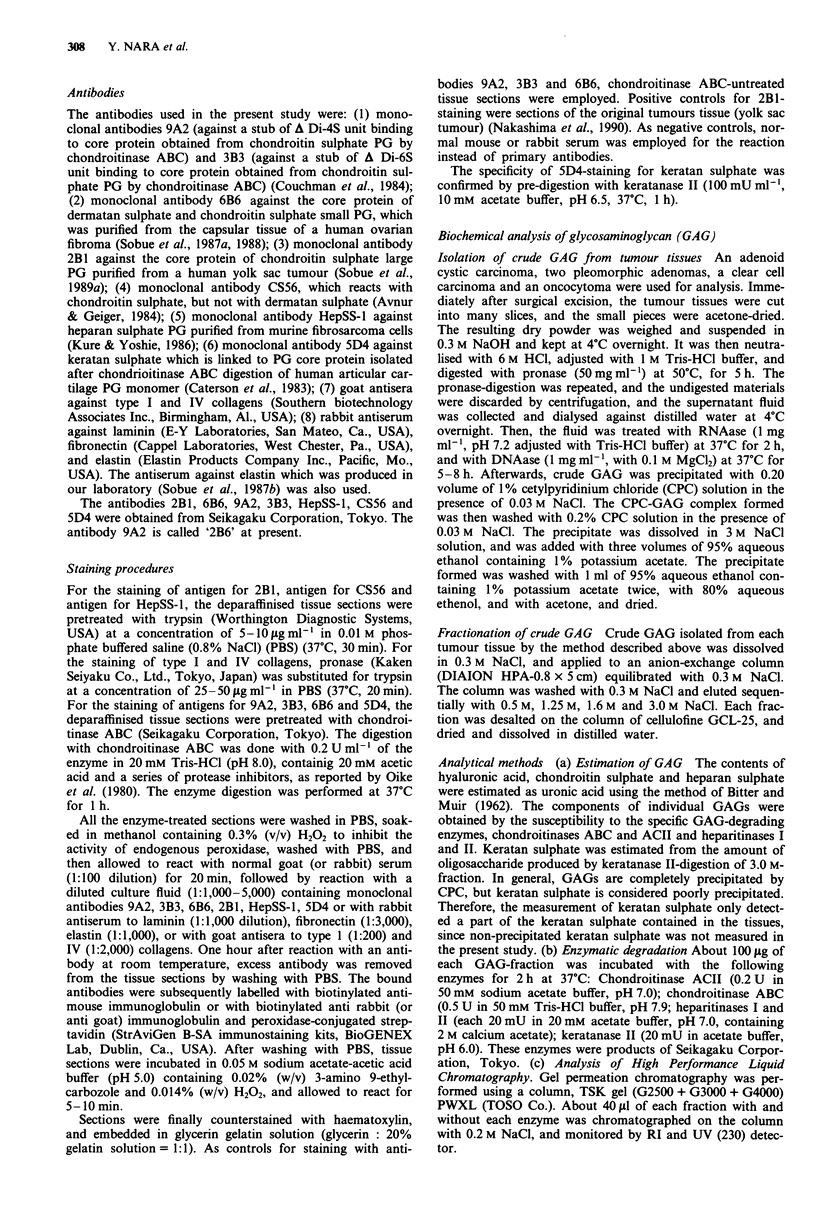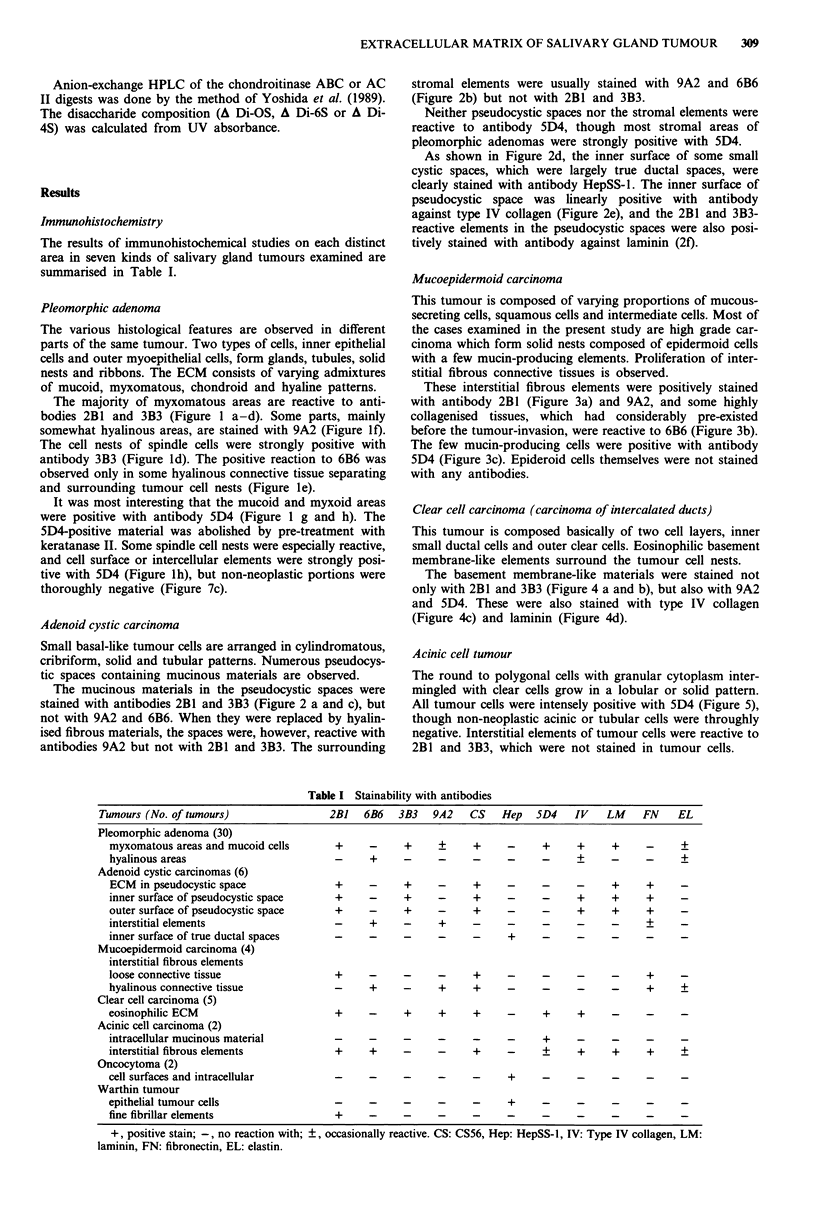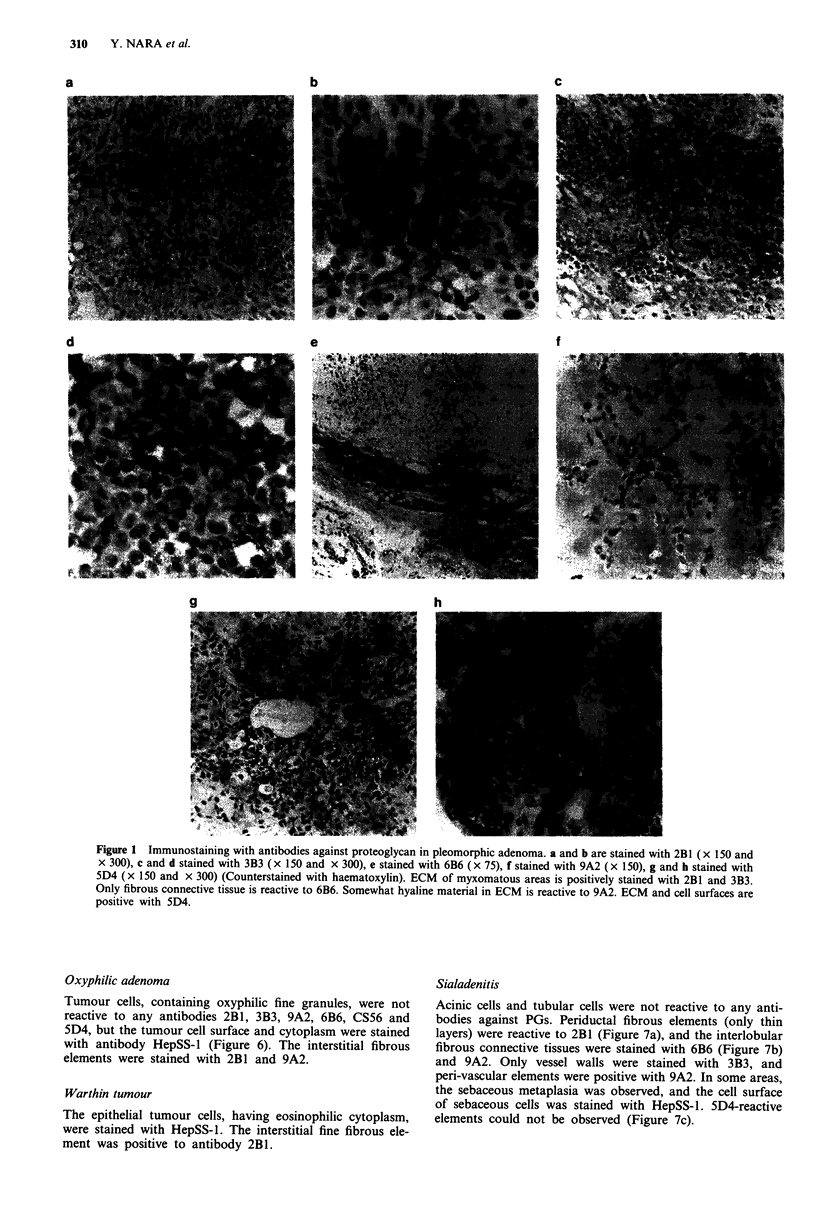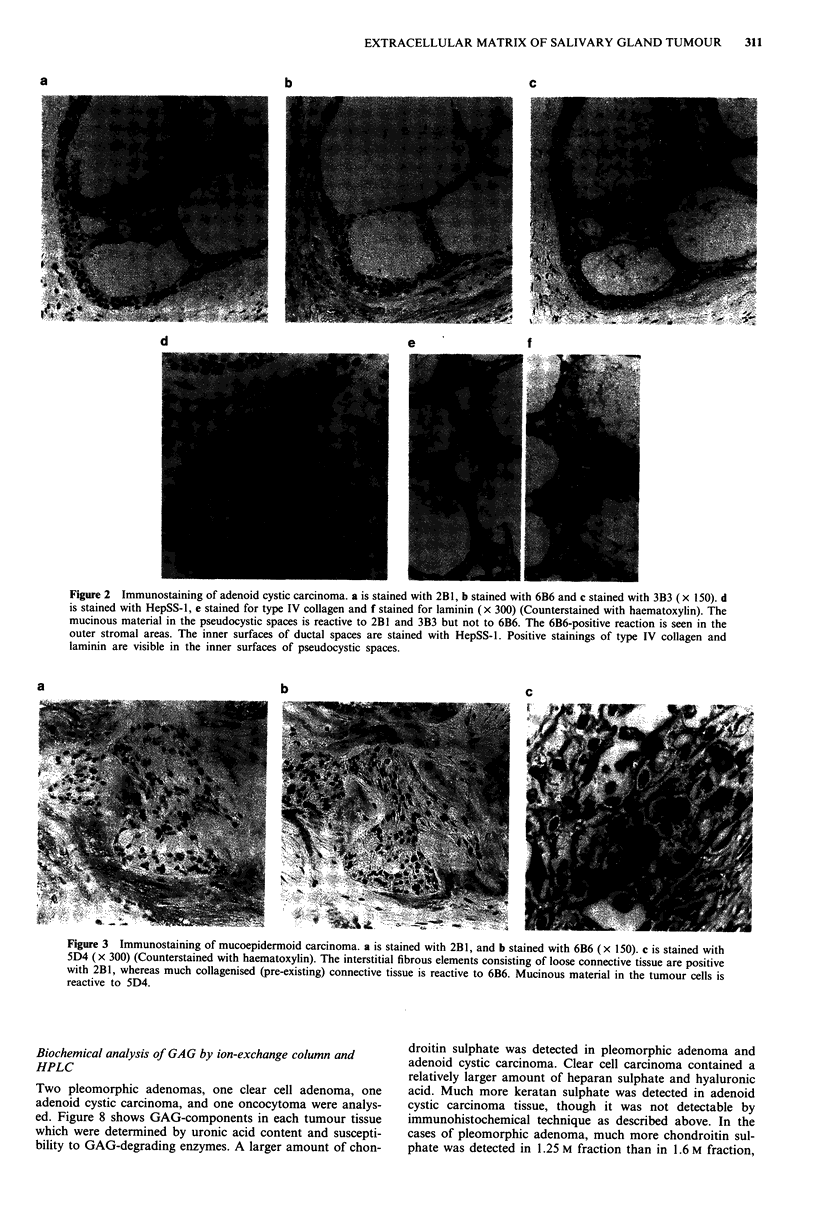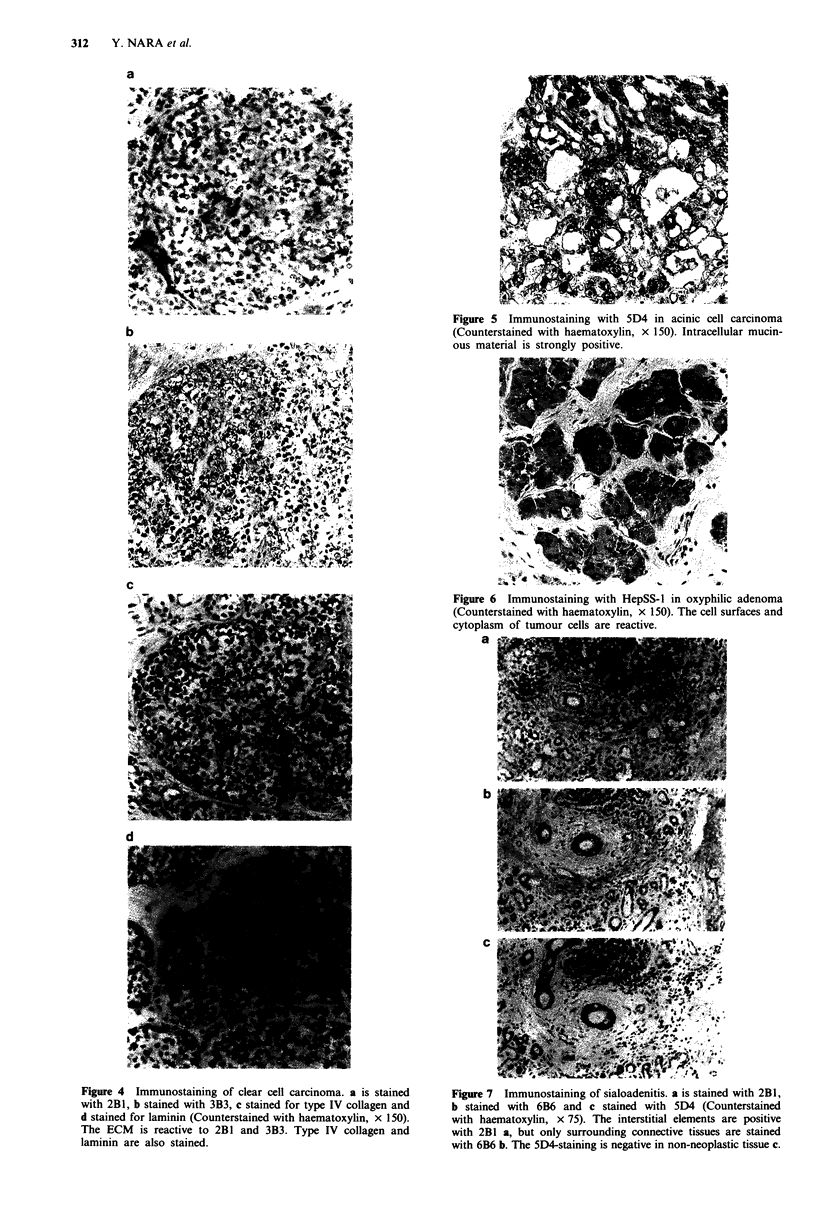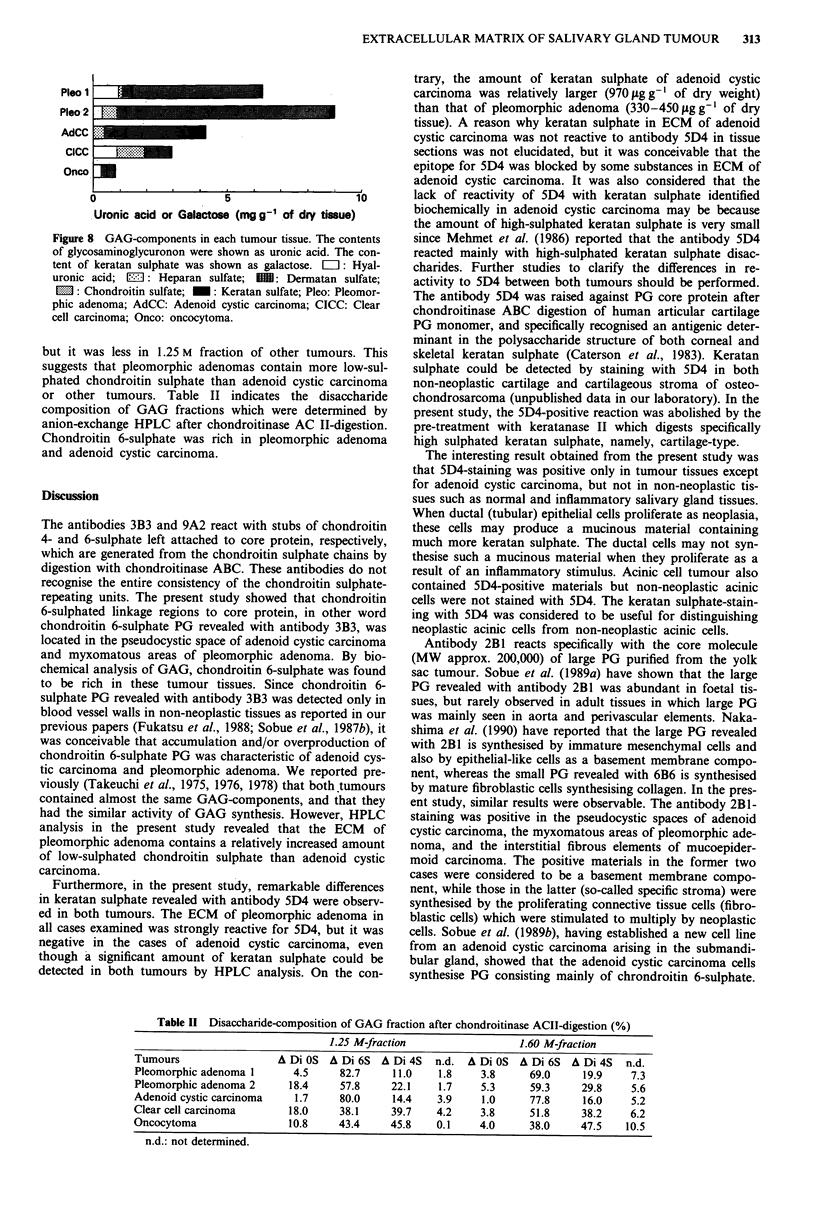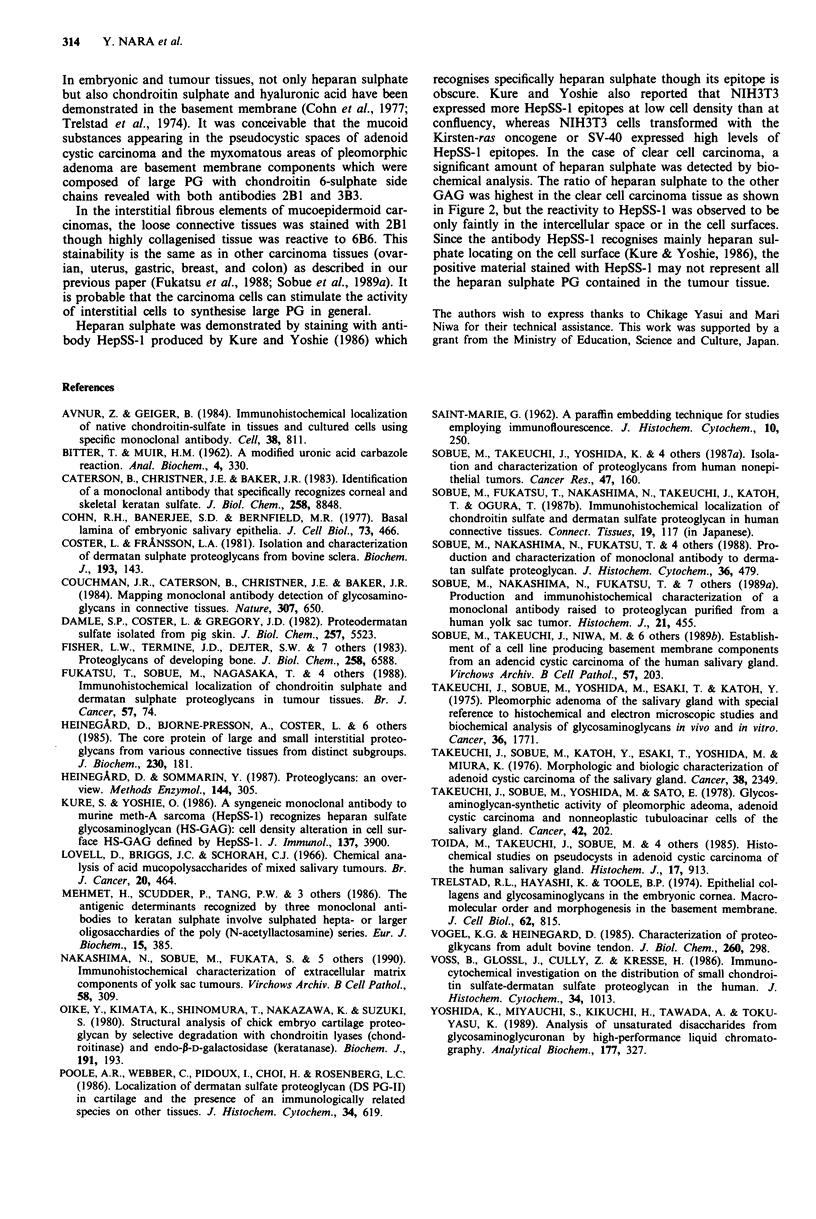# Immunohistochemical characterisation of extracellular matrix components of salivary gland tumours.

**DOI:** 10.1038/bjc.1991.297

**Published:** 1991-08

**Authors:** Y. Nara, J. Takeuchi, K. Yoshida, T. Fukatsu, T. Nagasaka, T. Kawaguchi, N. Meng, H. Kikuchi, N. Nakashima

**Affiliations:** Department of Laboratory Medicine, Nagoya University School of Medicine, Japan.

## Abstract

**Images:**


Br. J. Cancer (1991), 64, 307-314 © Macmillan Press Ltd., 1991

### Immunohistochemical characterisation of extracellular matrix components of salivary gland tumours

Y. Nara[1], J. Takeuchi[1,2], K. Yoshida[3], T. Fukatsu[1], T. Nagasaka[2], T. Kawaguchi[1], N. Meng[1], H. Kikuchi[3] & N. Nakashima[1]

[1]*Department of Laboratory Medicine, Nagoya University School of Medicine and* [2]*Division of Pathology, Clinical Laboratory, Nagoya University Hospital; 65 Tsurumai-cho, Showa-ku, Nagoya 466; and* [3]*Tokyo Research Institute, Seikagaku Corporation, Tokyo 189, Japan.*

**Summary** Proteoglycans (PGs) were localised immunohistochemically in 52 salivary gland tumours including pleomorphic adenoma, adenoid cystic carcinoma, acinic cell carcinoma, oncocytoma, mucoepidermoid carcinoma, clear cell tumour and Warthin tumour, using antibodies raised against large PG, small PG, chondroitin 4-sulphate PG, chondroitin 6-sulphate PG, heparan sulphate PG and keratan sulphate PG. Large PGs were mainly observed in mucinous materials of extracellular matrix (ECM) and interstitial fibrous element of tumour tissues, while small PGs were located only in hyaline matrix and surrounding fibrous (capsular) connective tissues. Chondroitin 6-sulphate PG was detected in the ECM of pleomorphic adenomas and clear cell carcinomas and in pseudocystic spaces of adenoid cystic carcinomas, but only in vessel walls in non-neoplastic tissues. Keratan sulphate PG was observed to locate in mucinous material of pleomorphic adenomas, acinic cell carcinomas and clear cell carcinomas, but not in the adenoid cystic carcinomas examined, and it was also unobservable in non-neoplastic salivary gland tissues. Heparan sulphate PG was observed on the inner surfaces of true ductal spaces of adenoid cystic carcinomas and on cell surfaces of oncocytoma cells. By HPLC analysis, individual glycosaminoglycans contained in tumour tissues were compared. Chondroitin 6-sulphate PG was very rich in ECM of pleomorphic adenomas and adenoid cystic carcinomas. Pleomorphic adenomas contained relatively more low-sulphated chondroitin sulphate than adenoid cystic carcinomas and other tumours.

The most characteristic feature of salivary gland tumours is the presence of a significant amount of extracellular matrix (ECM) consisting of proteoglycan (PG) and other glycoproteins. It is well known that the myxomatous areas in pleomorphic adenoma contain a large amount of mucinous substances which are mainly composed of PG. Adenoid cystic carcinomas characteristically shows many pseudocystic spaces which also contain a large amount of PG. Biochemical analysis and histochemical studies of ECM components in the salivary gland tumours have been performed (Lovell *et al.*, 1966; Takeuchi *et al.*, 1975, 1976, 1978; Toida *et al.*, 1985), but the precise localisation of individual PG-components have not been thoroughly elucidated.

PGs are macromolecules composed of a core protein to which one or more glycosaminoglycan (GAG) side chains are covalently linked. These molecules are major components of the ECM and comprise a large family, in which individual differences can be noted in the chemical nature of core proteins and GAG-side chains. Recently, PGs have been divided into two families, small and large (Heinegård & Sommarin, 1987). The small PGs, consisting of Mr 43,000–45,000 core molecules, have been purified from various tissues (Coster & Frånson, 1981; Damle *et al.*, 1982; Vogel & Heinegård *et al.*, 1985; Sobue *et al.*, 1987*a*). Monoclonal antibodies have been produced against the small PG by a number of investigators (Poole *et al.*, 1986; Voss *et al.*, 1986; Sobue *et al.*, 1988). The immunohistochemical localisation of the small PG families has been widely noted in the ECM of various human tissues (Sobue *et al.*, 1987*b* 1988). As for the large PG family, non-aggregating large chondroitin sulphate PG with Mr of $1 \times 10^6$–$2.5 \times 10^6$ has been detected in metaphyses, skeletal muscle, skin and tumour tissues (Fisher *et al.*, 1983; Heinegård *et al.*, 1985; Sobue *et al.*, 1987*a*, 1989*a*).

Recently, we produced a monoclonal antibody (2B1) raised against large PG purified from a human yolk sac tumour (Sobue *et al.*, 1989*a*), and monoclonal antibody 6B6, which reacts specifically with small PG purified from ovarian fibroma capsule (Sobue *et al.*, 1988). Couchman *et al.* (1984) produced antibodies 9A2 and 3B3 which were raised against a stub of Δ Di-4S and Δ Di-6S unit binding to a core protein by a linkage tetrasaccharide obtained from chondroitin sulphate PG by chondroitinase ABC-treatment. Using these antibodies, Couchman *et al.* (1984) demonstrated immunohistochemically a specific and distinctive distribution of chondroitin 4- or 6-sulphate PG in different connective tissues.

In the present study, in order to clarify the histogenesis and biological characteristics of each salivary gland tumour, ECM components produced by tumour cells were characterised immunohistochemically using the antibodies against large PG, small PG, chondroitin 4- or 6-sulphate PG, heparan sulphate PG and keratan sulphate PG. The specific and distinctive ECM components were found to be characteristic of each type of salivary gland tumour. Individual GAGs were also examined in some tumour tissues by HPLC analysis after digestion with GAG-degrading enzymes.

### Materials and methods

### *Tissues*

The salivary gland tumour tissues examined in the present study were: 30 pleomorphic adenomas, six adenoid cycstic carcinomas, five clear cell tumours, four mucoepidermoid tumours, two acinic cell tumours, three papillary cystadenoma lymphomatosum (Warthin tumour) and two oxyphilic adenomas (oncocytomas). Three cases of chronic sialadenitis were also investigated. These specimens were obtained from biopsies at Nagoya University Hospital. Tissue slices approximately 3 mm thick were cut from the excised tissues, and fixed in glacial acetic acid 1% (v/v) in 95% ethanol (Sainte-Marie, 1962) at 4°C for 12–24 h. The specimens were then dehydrated in an ethanol series of ascending concentrations, embedded in paraffin wax, and sectioned at a thickness of 4 μm.

Correspondence: Y. Nara.

Abbreviations: Δ Di-6S, 2-acetamide-2-deoxyl-3-*O*-(β-D-gluco-4-enepyranosyluronic acid)-6-*O*-sulfo-D-galactose; Δ Di-4S, 2-acetamide-2-deoxyl-3-*O*-(β-D-gluco-4-enepyranosyluronic acid)-4-*O*-sulfo-D-galactose; Δ Di-OS, 2-acetamide-2-deoxyl-3-*O*-(β-D-gluco-4-enopyranosyluronic acid)-D-galactose.

Received 27 November 1990; and in revised form 4 April 1991.

*Antibodies*

The antibodies used in the present study were: (1) monoclonal antibodies 9A2 (against a stub of Δ Di-4S unit binding to core protein obtained from chondroitin sulphate PG by chondroitinase ABC) and 3B3 (against a stub of Δ Di-6S unit binding to core protein obtained from chondroitin sulphate PG by chondroitinase ABC) (Couchman *et al.*, 1984); (2) monoclonal antibody 6B6 against the core protein of dermatan sulphate and chondroitin sulphate small PG, which was purified from the capsular tissue of a human ovarian fibroma (Sobue *et al.*, 1987*a*, 1988); (3) monoclonal antibody 2B1 against the core protein of chondroitin sulphate large PG purified from a human yolk sac tumour (Sobue *et al.*, 1989*a*); (4) monoclonal antibody CS56, which reacts with chondroitin sulphate, but not with dermatan sulphate (Avnur & Geiger, 1984); (5) monoclonal antibody HepSS-1 against heparan sulphate PG purified from murine fibrosarcoma cells (Kure & Yoshie, 1986); (6) monoclonal antibody 5D4 against keratan sulphate which is linked to PG core protein isolated after chondrioitinase ABC digestion of human articular cartilage PG monomer (Caterson *et al.*, 1983); (7) goat antisera against type I and IV collagens (Southern biotechnology Associates Inc., Birmingham, Al., USA); (8) rabbit antiserum against laminin (E-Y Laboratories, San Mateo, Ca., USA), fibronectin (Cappel Laboratories, West Chester, Pa., USA), and elastin (Elastin Products Company Inc., Pacific, Mo., USA). The antiserum against elastin which was produced in our laboratory (Sobue *et al.*, 1987*b*) was also used.

The antibodies 2B1, 6B6, 9A2, 3B3, HepSS-1, CS56 and 5D4 were obtained from Seikagaku Corporation, Tokyo. The antibody 9A2 is called '2B6' at present.

*Staining procedures*

For the staining of antigen for 2B1, antigen for CS56 and antigen for HepSS-1, the deparaffinised tissue sections were pretreated with trypsin (Worthington Diagnostic Systems, USA) at a concentration of 5–10 $\mu g\ ml^{-1}$ in 0.01 M phosphate buffered saline (0.8% NaCl) (PBS) (37°C, 30 min). For the staining of type I and IV collagens, pronase (Kaken Seiyaku Co., Ltd., Tokyo, Japan) was substituted for trypsin at a concentration of 25–50 $\mu g\ ml^{-1}$ in PBS (37°C, 20 min). For the staining of antigens for 9A2, 3B3, 6B6 and 5D4, the deparaffinised tissue sections were pretreated with chondroitinase ABC (Seikagaku Corporation, Tokyo). The digestion with chondroitinase ABC was done with 0.2 $U\ ml^{-1}$ of the enzyme in 20 mM Tris-HCl (pH 8.0), containig 20 mM acetic acid and a series of protease inhibitors, as reported by Oike *et al.* (1980). The enzyme digestion was performed at 37°C for 1 h.

All the enzyme-treated sections were washed in PBS, soaked in methanol containing 0.3% (v/v) $H_2O_2$ to inhibit the activity of endogenous peroxidase, washed with PBS, and then allowed to react with normal goat (or rabbit) serum (1:100 dilution) for 20 min, followed by reaction with a diluted culture fluid (1:1,000–5,000) containing monoclonal antibodies 9A2, 3B3, 6B6, 2B1, HepSS-1, 5D4 or with rabbit antiserum to laminin (1:1,000 dilution), fibronectin (1:3,000), elastin (1:1,000), or with goat antisera to type 1 (1:200) and IV (1:2,000) collagens. One hour after reaction with an antibody at room temperature, excess antibody was removed from the tissue sections by washing with PBS. The bound antibodies were subsequently labelled with biotinylated anti-mouse immunoglobulin or with biotinylated anti rabbit (or anti goat) immunoglobulin and peroxidase-conjugated streptavidin (StrAviGen B-SA immunostaining kits, BioGENEX Lab, Dublin, Ca., USA). After washing with PBS, tissue sections were incubated in 0.05 M sodium acetate-acetic acid buffer (pH 5.0) containing 0.02% (w/v) 3-amino 9-ethyl-carbozole and 0.014% (w/v) $H_2O_2$, and allowed to react for 5–10 min.

Sections were finally counterstained with haematoxylin, and embedded in glycerin gelatin solution (glycerin : 20% gelatin solution = 1:1). As controls for staining with antibodies 9A2, 3B3 and 6B6, chondroitinase ABC-untreated tissue sections were employed. Positive controls for 2B1-staining were sections of the original tumours tissue (yolk sac tumour) (Nakashima *et al.*, 1990). As negative controls, normal mouse or rabbit serum was employed for the reaction instead of primary antibodies.

The specificity of 5D4-staining for keratan sulphate was confirmed by pre-digestion with keratanase II (100 $mU\ ml^{-1}$, 10 mM acetate buffer, pH 6.5, 37°C, 1 h).

*Biochemical analysis of glycosaminoglycan (GAG)*

*Isolation of crude GAG from tumour tissues* An adenoid cystic carcinoma, two pleomorphic adenomas, a clear cell carcinoma and an oncocytoma were used for analysis. Immediately after surgical excision, the tumour tissues were cut into many slices, and the small pieces were acetone-dried. The resulting dry powder was weighed and suspended in 0.3 M NaOH and kept at 4°C overnight. It was then neutralised with 6 M HCl, adjusted with 1 M Tris-HCl buffer, and digested with pronase (50 $mg\ ml^{-1}$) at 50°C, for 5 h. The pronase-digestion was repeated, and the undigested materials were discarded by centrifugation, and the supernatant fluid was collected and dialysed against distilled water at 4°C overnight. Then, the fluid was treated with RNAase (1 mg $ml^{-1}$, pH 7.2 adjusted with Tris-HCl buffer) at 37°C for 2 h, and with DNAase (1 $mg\ ml^{-1}$, with 0.1 M $MgCl_2$) at 37°C for 5–8 h. Afterwards, crude GAG was precipitated with 0.20 volume of 1% cetylpyridinium chloride (CPC) solution in the presence of 0.03 M NaCl. The CPC-GAG complex formed was then washed with 0.2% CPC solution in the presence of 0.03 M NaCl. The precipitate was dissolved in 3 M NaCl solution, and was added with three volumes of 95% aqueous ethanol containing 1% potassium acetate. The precipitate formed was washed with 1 ml of 95% aqueous ethanol containing 1% potassium acetate twice, with 80% aqueous ethenol, and with acetone, and dried.

*Fractionation of crude GAG* Crude GAG isolated from each tumour tissue by the method described above was dissolved in 0.3 M NaCl, and applied to an anion-exchange column (DIAION HPA-0.8 × 5 cm) equilibrated with 0.3 M NaCl. The column was washed with 0.3 M NaCl and eluted sequentially with 0.5 M, 1.25 M, 1.6 M and 3.0 M NaCl. Each fraction was desalted on the column of cellulofine GCL-25, and dried and dissolved in distilled water.

*Analytical methods* (a) *Estimation of GAG* The contents of hyaluronic acid, chondroitin sulphate and heparan sulphate were estimated as uronic acid using the method of Bitter and Muir (1962). The components of individual GAGs were obtained by the susceptibility to the specific GAG-degrading enzymes, chondroitinases ABC and ACII and heparitinases I and II. Keratan sulphate was estimated from the amount of oligosaccharide produced by keratanase II-digestion of 3.0 M-fraction. In general, GAGs are completely precipitated by CPC, but keratan sulphate is considered poorly precipitated. Therefore, the measurement of keratan sulphate only detected a part of the keratan sulphate contained in the tissues, since non-precipitated keratan sulphate was not measured in the present study. (b) *Enzymatic degradation* About 100 $\mu g$ of each GAG-fraction was incubated with the following enzymes for 2 h at 37°C: Chondroitinase ACII (0.2 U in 50 mM sodium acetate buffer, pH 7.0); chondroitinase ABC (0.5 U in 50 mM Tris-HCl buffer, pH 7.9; heparitinases I and II (each 20 mU in 20 mM acetate buffer, pH 7.0, containing 2 M calcium acetate); keratanase II (20 mU in acetate buffer, pH 6.0). These enzymes were products of Seikagaku Corporation, Tokyo. (c) *Analysis of High Performance Liquid Chromatography.* Gel permeation chromatography was performed using a column, TSK gel (G2500 + G3000 + G4000) PWXL (TOSO Co.). About 40 $\mu l$ of each fraction with and without each enzyme was chromatographed on the column with 0.2 M NaCl, and monitored by RI and UV (230) detector.

Anion-exchange HPLC of the chondroitinase ABC or AC II digests was done by the method of Yoshida *et al.* (1989). The disaccharide composition (Δ Di-OS, Δ Di-6S or Δ Di-4S) was calculated from UV absorbance.

### Results

### *Immunohistochemistry*

The results of immunohistochemical studies on each distinct area in seven kinds of salivary gland tumours examined are summarised in Table I.

### *Pleomorphic adenoma*

The various histological features are observed in different parts of the same tumour. Two types of cells, inner epithelial cells and outer myoepithelial cells, form glands, tubules, solid nests and ribbons. The ECM consists of varying admixtures of mucoid, myxomatous, chondroid and hyaline patterns.

The majority of myxomatous areas are reactive to antibodies 2B1 and 3B3 (Figure 1 a–d). Some parts, mainly somewhat hyalinous areas, are stained with 9A2 (Figure 1f). The cell nests of spindle cells were strongly positive with antibody 3B3 (Figure 1d). The positive reaction to 6B6 was observed only in some hyalinous connective tissue separating and surrounding tumour cell nests (Figure 1e).

It was most interesting that the mucoid and myxoid areas were positive with antibody 5D4 (Figure 1 g and h). The 5D4-positive material was abolished by pre-treatment with keratanase II. Some spindle cell nests were especially reactive, and cell surface or intercellular elements were strongly positive with 5D4 (Figure 1h), but non-neoplastic portions were thoroughly negative (Figure 7c).

### *Adenoid cystic carcinoma*

Small basal-like tumour cells are arranged in cylindromatous, cribriform, solid and tubular patterns. Numerous pseudocystic spaces containing mucinous materials are observed.

The mucinous materials in the pseudocystic spaces were stained with antibodies 2B1 and 3B3 (Figure 2 a and c), but not with 9A2 and 6B6. When they were replaced by hyalinised fibrous materials, the spaces were, however, reactive with antibodies 9A2 but not with 2B1 and 3B3. The surrounding stromal elements were usually stained with 9A2 and 6B6 (Figure 2b) but not with 2B1 and 3B3.

Neither pseudocystic spaces nor the stromal elements were reactive to antibody 5D4, though most stromal areas of pleomorphic adenomas were strongly positive with 5D4.

As shown in Figure 2d, the inner surface of some small cystic spaces, which were largely true ductal spaces, were clearly stained with antibody HepSS-1. The inner surface of pseudocystic space was linearly positive with antibody against type IV collagen (Figure 2e), and the 2B1 and 3B3-reactive elements in the pseudocystic spaces were also positively stained with antibody against laminin (2f).

### *Mucoepidermoid carcinoma*

This tumour is composed of varying proportions of mucous-secreting cells, squamous cells and intermediate cells. Most of the cases examined in the present study are high grade carcinoma which form solid nests composed of epidermoid cells with a few mucin-producing elements. Proliferation of interstitial fibrous connective tissues is observed.

These interstitial fibrous elements were positively stained with antibody 2B1 (Figure 3a) and 9A2, and some highly collagenised tissues, which had considerably pre-existed before the tumour-invasion, were reactive to 6B6 (Figure 3b). The few mucin-producing cells were positive with antibody 5D4 (Figure 3c). Epideroid cells themselves were not stained with any antibodies.

### *Clear cell carcinoma (carcinoma of intercalated ducts)*

This tumour is composed basically of two cell layers, inner small ductal cells and outer clear cells. Eosinophilic basement membrane-like elements surround the tumour cell nests.

The basement membrane-like materials were stained not only with 2B1 and 3B3 (Figure 4 a and b), but also with 9A2 and 5D4. These were also stained with type IV collagen (Figure 4c) and laminin (Figure 4d).

### *Acinic cell tumour*

The round to polygonal cells with granular cytoplasm intermingled with clear cells grow in a lobular or solid pattern. All tumour cells were intensely positive with 5D4 (Figure 5), though non-neoplastic acinic or tubular cells were throughly negative. Interstitial elements of tumour cells were reactive to 2B1 and 3B3, which were not stained in tumour cells.

**Table I** Stainability with antibodies

| *Tumours (No. of tumours)* | *2B1* | *6B6* | *3B3* | *9A2* | *CS* | *Hep* | *5D4* | *IV* | *LM* | *FN* | *EL* |
|---|---|---|---|---|---|---|---|---|---|---|---|
| Pleomorphic adenoma (30) | | | | | | | | | | | |
| myxomatous areas and mucoid cells | + | – | + | ± | + | – | + | + | + | – | ± |
| hyalinous areas | – | + | – | – | – | – | – | ± | – | – | ± |
| Adenoid cystic carcinomas (6) | | | | | | | | | | | |
| ECM in pseudocystic space | + | – | + | – | + | – | – | – | + | + | – |
| inner surface of pseudocystic space | + | – | + | – | + | – | – | + | + | + | – |
| outer surface of pseudocystic space | + | – | + | – | + | – | – | + | + | + | – |
| interstitial elements | – | + | – | + | – | – | – | – | – | ± | – |
| inner surface of true ductal spaces | – | – | – | – | – | + | – | – | – | – | – |
| Mucoepidermoid carcinoma (4) | | | | | | | | | | | |
| interstitial fibrous elements | | | | | | | | | | | |
| loose connective tissue | + | – | – | – | + | – | – | – | – | + | – |
| hyalinous connective tissue | – | + | – | + | + | – | – | – | – | + | ± |
| Clear cell carcinoma (5) | | | | | | | | | | | |
| eosinophilic ECM | + | – | + | + | + | – | + | + | – | – | – |
| Acinic cell carcinoma (2) | | | | | | | | | | | |
| intracellular mucinous material | – | – | – | – | – | – | + | – | – | – | – |
| interstitial fibrous elements | + | + | – | – | + | – | ± | + | + | + | ± |
| Oncocytoma (2) | | | | | | | | | | | |
| cell surfaces and intracellular | – | – | – | – | – | + | – | – | – | – | – |
| Warthin tumour | | | | | | | | | | | |
| epithelial tumour cells | – | – | – | – | – | + | – | – | – | – | – |
| fine fibrillar elements | + | – | – | – | – | – | – | – | – | – | – |

+, positive stain; –, no reaction with; ±, occasionally reactive. CS: CS56, Hep: HepSS-1, IV: Type IV collagen, LM: laminin, FN: fibronectin, EL: elastin.

**Figure 1** Immunostaining with antibodies against proteoglycan in pleomorphic adenoma. **a** and **b** are stained with 2B1 (× 150 and × 300), **c** and **d** stained with 3B3 (× 150 and × 300), **e** stained with 6B6 (× 75), **f** stained with 9A2 (× 150), **g** and **h** stained with 5D4 (× 150 and × 300) (Counterstained with haematoxylin). ECM of myxomatous areas is positively stained with 2B1 and 3B3. Only fibrous connective tissue is reactive to 6B6. Somewhat hyaline material in ECM is reactive to 9A2. ECM and cell surfaces are positive with 5D4.

*Oxyphilic adenoma*

Tumour cells, containing oxyphilic fine granules, were not reactive to any antibodies 2B1, 3B3, 9A2, 6B6, CS56 and 5D4, but the tumour cell surface and cytoplasm were stained with antibody HepSS-1 (Figure 6). The interstitial fibrous elements were stained with 2B1 and 9A2.

*Warthin tumour*

The epithelial tumour cells, having eosinophilic cytoplasm, were stained with HepSS-1. The interstitial fine fibrous element was positive to antibody 2B1.

*Sialadenitis*

Acinic cells and tubular cells were not reactive to any antibodies against PGs. Periductal fibrous elements (only thin layers) were reactive to 2B1 (Figure 7a), and the interlobular fibrous connective tissues were stained with 6B6 (Figure 7b) and 9A2. Only vessel walls were stained with 3B3, and peri-vascular elements were positive with 9A2. In some areas, the sebaceous metaplasia was observed, and the cell surface of sebaceous cells was stained with HepSS-1. 5D4-reactive elements could not be observed (Figure 7c).

**Figure 2** Immunostaining of adenoid cystic carcinoma. **a** is stained with 2B1, **b** stained with 6B6 and **c** stained with 3B3 (× 150). **d** is stained with HepSS-1, **e** stained for type IV collagen and **f** stained for laminin (× 300) (Counterstained with haematoxylin). The mucinous material in the pseudocystic spaces is reactive to 2B1 and 3B3 but not to 6B6. The 6B6-positive reaction is seen in the outer stromal areas. The inner surfaces of ductal spaces are stained with HepSS-1. Positive stainings of type IV collagen and laminin are visible in the inner surfaces of pseudocystic spaces.

**Figure 3** Immunostaining of mucoepidermoid carcinoma. **a** is stained with 2B1, and **b** stained with 6B6 (× 150). **c** is stained with 5D4 (× 300) (Counterstained with haematoxylin). The interstitial fibrous elements consisting of loose connective tissue are positive with 2B1, whereas much collagenised (pre-existing) connective tissue is reactive to 6B6. Mucinous material in the tumour cells is reactive to 5D4.

*Biochemical analysis of GAG by ion-exchange column and HPLC*

Two pleomorphic adenomas, one clear cell adenoma, one adenoid cystic carcinoma, and one oncocytoma were analysed. Figure 8 shows GAG-components in each tumour tissue which were determined by uronic acid content and susceptibility to GAG-degrading enzymes. A larger amount of chondroitin sulphate was detected in pleomorphic adenoma and adenoid cystic carcinoma. Clear cell carcinoma contained a relatively larger amount of heparan sulphate and hyaluronic acid. Much more keratan sulphate was detected in adenoid cystic carcinoma tissue, though it was not detectable by immunohistochemical technique as described above. In the cases of pleomorphic adenoma, much more chondroitin sulphate was detected in 1.25 M fraction than in 1.6 M fraction,

**Figure 4** Immunostaining of clear cell carcinoma. **a** is stained with 2B1, **b** stained with 3B3, **c** stained for type IV collagen and **d** stained for laminin (Counterstained with haematoxylin, × 150). The ECM is reactive to 2B1 and 3B3. Type IV collagen and laminin are also stained.

**Figure 5** Immunostaining with 5D4 in acinic cell carcinoma (Counterstained with haematoxylin, × 150). Intracellular mucinous material is strongly positive.

**Figure 6** Immunostaining with HepSS-1 in oxyphilic adenoma (Counterstained with haematoxylin, × 150). The cell surfaces and cytoplasm of tumour cells are reactive.

**Figure 7** Immunostaining of sialoadenitis. **a** is stained with 2B1, **b** stained with 6B6 and **c** stained with 5D4 (Counterstained with haematoxylin, × 75). The interstitial elements are positive with 2B1 **a**, but only surrounding connective tissues are stained with 6B6 **b**. The 5D4-staining is negative in non-neoplastic tissue **c**.

**Figure 8** GAG-components in each tumour tissue. The contents of glycosaminoglycuronon were shown as uronic acid. The content of keratan sulphate was shown as galactose. □ : Hyaluronic acid; ▦ : Heparan sulfate; ▩ : Dermatan sulfate; ▤ : Chondroitin sulfate; ■ : Keratan sulfate; Pleo: Pleomorphic adenoma; AdCC: Adenoid cystic carcinoma; CICC: Clear cell carcinoma; Onco: oncocytoma.

but it was less in 1.25 M fraction of other tumours. This suggests that pleomorphic adenomas contain more low-sulphated chondroitin sulphate than adenoid cystic carcinoma or other tumours. Table II indicates the disaccharide composition of GAG fractions which were determined by anion-exchange HPLC after chondroitinase AC II-digestion. Chondroitin 6-sulphate was rich in pleomorphic adenoma and adenoid cystic carcinoma.

### Discussion

The antibodies 3B3 and 9A2 react with stubs of chondroitin 4- and 6-sulphate left attached to core protein, respectively, which are generated from the chondroitin sulphate chains by digestion with chondroitinase ABC. These antibodies do not recognise the entire consistency of the chondroitin sulphate-repeating units. The present study showed that chondroitin 6-sulphated linkage regions to core protein, in other word chondroitin 6-sulphate PG revealed with antibody 3B3, was located in the pseudocystic space of adenoid cystic carcinoma and myxomatous areas of pleomorphic adenoma. By biochemical analysis of GAG, chondroitin 6-sulphate was found to be rich in these tumour tissues. Since chondroitin 6-sulphate PG revealed with antibody 3B3 was detected only in blood vessel walls in non-neoplastic tissues as reported in our previous papers (Fukatsu *et al.*, 1988; Sobue *et al.*, 1987*b*), it was conceivable that accumulation and/or overproduction of chondroitin 6-sulphate PG was characteristic of adenoid cystic carcinoma and pleomorphic adenoma. We reported previously (Takeuchi *et al.*, 1975, 1976, 1978) that both tumours contained almost the same GAG-components, and that they had the similar activity of GAG synthesis. However, HPLC analysis in the present study revealed that the ECM of pleomorphic adenoma contains a relatively increased amount of low-sulphated chondroitin sulphate than adenoid cystic carcinoma.

Furthermore, in the present study, remarkable differences in keratan sulphate revealed with antibody 5D4 were observed in both tumours. The ECM of pleomorphic adenoma in all cases examined was strongly reactive for 5D4, but it was negative in the cases of adenoid cystic carcinoma, even though a significant amount of keratan sulphate could be detected in both tumours by HPLC analysis. On the contrary, the amount of keratan sulphate of adenoid cystic carcinoma was relatively larger (970 $\mu g\, g^{-1}$ of dry weight) than that of pleomorphic adenoma (330–450 $\mu g\, g^{-1}$ of dry tissue). A reason why keratan sulphate in ECM of adenoid cystic carcinoma was not reactive to antibody 5D4 in tissue sections was not elucidated, but it was conceivable that the epitope for 5D4 was blocked by some substances in ECM of adenoid cystic carcinoma. It was also considered that the lack of reactivity of 5D4 with keratan sulphate identified biochemically in adenoid cystic carcinoma may be because the amount of high-sulphated keratan sulphate is very small since Mehmet *et al.* (1986) reported that the antibody 5D4 reacted mainly with high-sulphated keratan sulphate disaccharides. Further studies to clarify the differences in reactivity to 5D4 between both tumours should be performed. The antibody 5D4 was raised against PG core protein after chondroitinase ABC digestion of human articular cartilage PG monomer, and specifically recognised an antigenic determinant in the polysaccharide structure of both corneal and skeletal keratan sulphate (Caterson *et al.*, 1983). Keratan sulphate could be detected by staining with 5D4 in both non-neoplastic cartilage and cartilageous stroma of osteochondrosarcoma (unpublished data in our laboratory). In the present study, the 5D4-positive reaction was abolished by the pre-treatment with keratanase II which digests specifically high sulphated keratan sulphate, namely, cartilage-type.

The interesting result obtained from the present study was that 5D4-staining was positive only in tumour tissues except for adenoid cystic carcinoma, but not in non-neoplastic tissues such as normal and inflammatory salivary gland tissues. When ductal (tubular) epithelial cells proliferate as neoplasia, these cells may produce a mucinous material containing much more keratan sulphate. The ductal cells may not synthesise such a mucinous material when they proliferate as a result of an inflammatory stimulus. Acinic cell tumour also contained 5D4-positive materials but non-neoplastic acinic cells were not stained with 5D4. The keratan sulphate-staining with 5D4 was considered to be useful for distinguishing neoplastic acinic cells from non-neoplastic acinic cells.

Antibody 2B1 reacts specifically with the core molecule (MW approx. 200,000) of large PG purified from the yolk sac tumour. Sobue *et al.* (1989*a*) have shown that the large PG revealed with antibody 2B1 was abundant in foetal tissues, but rarely observed in adult tissues in which large PG was mainly seen in aorta and perivascular elements. Nakashima *et al.* (1990) have reported that the large PG revealed with 2B1 is synthesised by immature mesenchymal cells and also by epithelial-like cells as a basement membrane component, whereas the small PG revealed with 6B6 is synthesised by mature fibroblastic cells synthesising collagen. In the present study, similar results were observable. The antibody 2B1-staining was positive in the pseudocystic spaces of adenoid cystic carcinoma, the myxomatous areas of pleomorphic adenoma, and the interstitial fibrous elements of mucoepidermoid carcinoma. The positive materials in the former two cases were considered to be a basement membrane component, while those in the latter (so-called specific stroma) were synthesised by the proliferating connective tissue cells (fibroblastic cells) which were stimulated to multiply by neoplastic cells. Sobue *et al.* (1989*b*), having established a new cell line from an adenoid cystic carcinoma arising in the submandibular gland, showed that the adenoid cystic carcinoma cells synthesise PG consisting mainly of chrondroitin 6-sulphate.

**Table II** Disaccharide-composition of GAG fraction after chondroitinase ACII-digestion (%)

| | *1.25 M-fraction* | | | | *1.60 M-fraction* | | | |
|---|---|---|---|---|---|---|---|---|
| Tumours | Δ Di 0S | Δ Di 6S | Δ Di 4S | n.d. | Δ Di 0S | Δ Di 6S | Δ Di 4S | n.d. |
| Pleomorphic adenoma 1 | 4.5 | 82.7 | 11.0 | 1.8 | 3.8 | 69.0 | 19.9 | 7.3 |
| Pleomorphic adenoma 2 | 18.4 | 57.8 | 22.1 | 1.7 | 5.3 | 59.3 | 29.8 | 5.6 |
| Adenoid cystic carcinoma | 1.7 | 80.0 | 14.4 | 3.9 | 1.0 | 77.8 | 16.0 | 5.2 |
| Clear cell carcinoma | 18.0 | 38.1 | 39.7 | 4.2 | 3.8 | 51.8 | 38.2 | 6.2 |
| Oncocytoma | 10.8 | 43.4 | 45.8 | 0.1 | 4.0 | 38.0 | 47.5 | 10.5 |

n.d.: not determined.

In embryonic and tumour tissues, not only heparan sulphate but also chondroitin sulphate and hyaluronic acid have been demonstrated in the basement membrane (Cohn et al., 1977; Trelstad et al., 1974). It was conceivable that the mucoid substances appearing in the pseudocystic spaces of adenoid cystic carcinoma and the myxomatous areas of pleomorphic adenoma are basement membrane components which were composed of large PG with chondroitin 6-sulphate side chains revealed with both antibodies 2B1 and 3B3.

In the interstitial fibrous elements of mucoepidermoid carcinomas, the loose connective tissues was stained with 2B1 though highly collagenised tissue was reactive to 6B6. This stainability is the same as in other carcinoma tissues (ovarian, uterus, gastric, breast, and colon) as described in our previous paper (Fukatsu et al., 1988; Sobue et al., 1989a). It is probable that the carcinoma cells can stimulate the activity of interstitial cells to synthesise large PG in general.

Heparan sulphate was demonstrated by staining with antibody HepSS-1 produced by Kure and Yoshie (1986) which recognises specifically heparan sulphate though its epitope is obscure. Kure and Yoshie also reported that NIH3T3 expressed more HepSS-1 epitopes at low cell density than at confluency, whereas NIH3T3 cells transformed with the Kirsten-*ras* oncogene or SV-40 expressed high levels of HepSS-1 epitopes. In the case of clear cell carcinoma, a significant amount of heparan sulphate was detected by biochemical analysis. The ratio of heparan sulphate to the other GAG was highest in the clear cell carcinoma tissue as shown in Figure 2, but the reactivity to HepSS-1 was observed to be only faintly in the intercellular space or in the cell surfaces. Since the antibody HepSS-1 recognises mainly heparan sulphate locating on the cell surface (Kure & Yoshie, 1986), the positive material stained with HepSS-1 may not represent all the heparan sulphate PG contained in the tumour tissue.

The authors wish to express thanks to Chikage Yasui and Mari Niwa for their technical assistance. This work was supported by a grant from the Ministry of Education, Science and Culture, Japan.

### References

AVNUR, Z. & GEIGER, B. (1984). Immunohistochemical localization of native chondroitin-sulfate in tissues and cultured cells using specific monoclonal antibody. *Cell*, **38**, 811.

BITTER, T. & MUIR, H.M. (1962). A modified uronic acid carbazole reaction. *Anal. Biochem.*, **4**, 330.

CATERSON, B., CHRISTNER, J.E. & BAKER, J.R. (1983). Identification of a monoclonal antibody that specifically recognizes corneal and skeletal keratan sulfate. *J. Biol. Chem.*, **258**, 8848.

COHN, R.H., BANERJEE, S.D. & BERNFIELD, M.R. (1977). Basal lamina of embryonic salivary epithelia. *J. Cell Biol.*, **73**, 466.

COSTER, L. & FRÅNSSON, L.A. (1981). Isolation and characterization of dermatan sulphate proteoglycans from bovine sclera. *Biochem. J.*, **193**, 143.

COUCHMAN, J.R., CATERSON, B., CHRISTNER, J.E. & BAKER, J.R. (1984). Mapping monoclonal antibody detection of glycosaminoglycans in connective tissues. *Nature*, **307**, 650.

DAMLE, S.P., COSTER, L. & GREGORY, J.D. (1982). Proteodermatan sulfate isolated from pig skin. *J. Biol. Chem.*, **257**, 5523.

FISHER, L.W., TERMINE, J.D., DEJTER, S.W. & 7 others (1983). Proteoglycans of developing bone. *J. Biol. Chem.*, **258**, 6588.

FUKATSU, T., SOBUE, M., NAGASAKA, T. & 4 others (1988). Immunohistochemical localization of chondroitin sulphate and dermatan sulphate proteoglycans in tumour tissues. *Br. J. Cancer*, **57**, 74.

HEINEGÅRD, D., BJORNE-PRESSON, A., COSTER, L. & 6 others (1985). The core protein of large and small interstitial proteoglycans from various connective tissues from distinct subgroups. *J. Biochem.*, **230**, 181.

HEINEGÅRD, D. & SOMMARIN, Y. (1987). Proteoglycans: an overview. *Methods Enzymol.*, **144**, 305.

KURE, S. & YOSHIE, O. (1986). A syngeneic monoclonal antibody to murine meth-A sarcoma (HepSS-1) recognizes heparan sulfate glycosaminoglycan (HS-GAG): cell density alteration in cell surface HS-GAG defined by HepSS-1. *J. Immunol.*, **137**, 3900.

LOVELL, D., BRIGGS, J.C. & SCHORAH, C.J. (1966). Chemical analysis of acid mucopolysaccharides of mixed salivary tumours. *Br. J. Cancer*, **20**, 464.

MEHMET, H., SCUDDER, P., TANG, P.W. & 3 others (1986). The antigenic determinants recognized by three monoclonal antibodies to keratan sulphate involve sulphated hepta- or larger oligosacchardies of the poly (N-acetyllactosamine) series. *Eur. J. Biochem.*, **15**, 385.

NAKASHIMA, N., SOBUE, M., FUKATA, S. & 5 others (1990). Immunohistochemical characterization of extracellular matrix components of yolk sac tumours. *Virchows Archiv. B Cell Pathol.*, **58**, 309.

OIKE, Y., KIMATA, K., SHINOMURA, T., NAKAZAWA, K. & SUZUKI, S. (1980). Structural analysis of chick embryo cartilage proteoglycan by selective degradation with chondroitin lyases (chondroitinase) and endo-β-D-galactosidase (keratanase). *Biochem. J.*, **191**, 193.

POOLE, A.R., WEBBER, C., PIDOUX, I., CHOI, H. & ROSENBERG, L.C. (1986). Localization of dermatan sulfate proteoglycan (DS PG-II) in cartilage and the presence of an immunologically related species on other tissues. *J. Histochem. Cytochem.*, **34**, 619.

SAINT-MARIE, G. (1962). A paraffin embedding technique for studies employing immunoflourescence. *J. Histochem. Cytochem.*, **10**, 250.

SOBUE, M., TAKEUCHI, J., YOSHIDA, K. & 4 others (1987*a*). Isolation and characterization of proteoglycans from human nonepithelial tumors. *Cancer Res.*, **47**, 160.

SOBUE, M., FUKATSU, T., NAKASHIMA, N., TAKEUCHI, J., KATOH, T. & OGURA, T. (1987b). Immunohistochemical localization of chondroitin sulfate and dermatan sulfate proteoglycan in human connective tissues. *Connect. Tissues*, **19**, 117 (in Japanese).

SOBUE, M., NAKASHIMA, N., FUKATSU, T. & 4 others (1988). Production and characterization of monoclonal antibody to dermatan sulfate proteoglycan. *J. Histochem. Cytochem.*, **36**, 479.

SOBUE, M., NAKASHIMA, N., FUKATSU, T. & 7 others (1989*a*). Production and immunohistochemical characterization of a monoclonal antibody raised to proteoglycan purified from a human yolk sac tumor. *Histochem. J.*, **21**, 455.

SOBUE, M., TAKEUCHI, J., NIWA, M. & 6 others (1989*b*). Establishment of a cell line producing basement membrane components from an adenoid cystic carcinoma of the human salivary gland. *Virchows Archiv. B Cell Pathol.*, **57**, 203.

TAKEUCHI, J., SOBUE, M., YOSHIDA, M., ESAKI, T. & KATOH, Y. (1975). Pleomorphic adenoma of the salivary gland with special reference to histochemical and electron microscopic studies and biochemical analysis of glycosaminoglycans *in vivo* and *in vitro*. *Cancer*, **36**, 1771.

TAKEUCHI, J., SOBUE, M., KATOH, Y., ESAKI, T., YOSHIDA, M. & MIURA, K. (1976). Morphologic and biologic characterization of adenoid cystic carcinoma of the salivary gland. *Cancer*, **38**, 2349.

TAKEUCHI, J., SOBUE, M., YOSHIDA, M. & SATO, E. (1978). Glycosaminoglycan-synthetic activity of pleomorphic adeoma, adenoid cystic carcinoma and nonneoplastic tubuloacinar cells of the salivary gland. *Cancer*, **42**, 202.

TOIDA, M., TAKEUCHI, J., SOBUE, M. & 4 others (1985). Histochemical studies on pseudocysts in adenoid cystic carcinoma of the human salivary gland. *Histochem. J.*, **17**, 913.

TRELSTAD, R.L., HAYASHI, K. & TOOLE, B.P. (1974). Epithelial collagens and glycosaminoglycans in the embryonic cornea. Macromolecular order and morphogenesis in the basement membrane. *J. Cell Biol.*, **62**, 815.

VOGEL, K.G. & HEINEGARD, D. (1985). Characterization of proteoglkycans from adult bovine tendon. *J. Biol. Chem.*, **260**, 298.

VOSS, B., GLOSSL, J., CULLY, Z. & KRESSE, H. (1986). Immunocytochemical investigation on the distribution of small chondroitin sulfate-dermatan sulfate proteoglycan in the human. *J. Histochem. Cytochem.*, **34**, 1013.

YOSHIDA, K., MIYAUCHI, S., KIKUCHI, H., TAWADA, A. & TOKUYASU, K. (1989). Analysis of unsaturated disaccharides from glycosaminoglycuronan by high-performance liquid chromatography. *Analytical Biochem.*, **177**, 327.